# The hepato-ovarian axis: genetic evidence for a causal association between non-alcoholic fatty liver disease and polycystic ovary syndrome

**DOI:** 10.1186/s12916-023-02775-0

**Published:** 2023-02-20

**Authors:** Dong Liu, Xue Gao, Xiong-Fei Pan, Tao Zhou, Cairong Zhu, Fei Li, Jian-Gao Fan, Giovanni Targher, Jian Zhao

**Affiliations:** 1 Ministry of Education and Shanghai Key Laboratory of Children’s Environmental Health, Xinhua Hospital, Shanghai Jiao Tong University School of Medicine, No.1665, Kongjiang Road, Yangpu District, Shanghai, 200092 China; 2 Department of Health Statistics, School of Public Health, Shanxi Medical University, Taiyuan, Shanxi China; 3 Ministry of Education Key Laboratory of Birth Defects and Related Diseases in Women and Children, West China Second University Hospital, Sichuan University, Chengdu, Sichuan China; 4 Shuangliu Institute of Women’s and Children’s Health, Shuangliu Maternal and Child Health Hospital, Chengdu, Sichuan China; 5 School of Public Health (Shenzhen), Shenzhen Campus of Sun Yat-sen University, Shenzhen, China; 6 West China School of Public Health and West China Fourth Hospital, Sichuan University, Chengdu, Sichuan China; 7 Department of Developmental and Behavioral Pediatric & Child Primary Care, Brain and Behavioral Research Unit of Shanghai Institute for Pediatric Research, Xinhua Hospital, Shanghai Jiao Tong University School of Medicine, Shanghai, China; 8 Department of Maternal and Child Health, School of Public Health, Shanghai Jiao Tong University, Shanghai, China; 9 Department of Gastroenterology, Xinhua Hospital, Shanghai Jiao Tong University School of Medicine, Shanghai, China; 10 Shanghai Key Lab of Pediatric Gastroenterology and Nutrition, Shanghai, China; 11 Section of Endocrinology, Diabetes and Metabolism, Department of Medicine, University of Verona, Verona, Italy; 12 MRC Integrative Epidemiology Unit, University of Bristol, Bristol, UK

**Keywords:** Non-alcoholic fatty liver disease, Polycystic ovary syndrome, Mendelian randomization, Fasting insulin, Sex hormones, Hepato-ovarian axis

## Abstract

**Background:**

Recent studies found associations between non-alcoholic fatty liver disease (NAFLD) and polycystic ovary syndrome (PCOS), but the causal nature of this association is still uncertain.

**Methods:**

We performed a bidirectional two-sample Mendelian randomization (MR) analysis to test for the causal association between NAFLD and PCOS using data from a large-scale biopsy-confirmed NAFLD genome-wide association study (GWAS) (1483 cases and 17,781 controls) and PCOS GWAS (10,074 cases and 103,164 controls) in European ancestries. Data from glycemic-related traits GWAS (in up to 200,622 individuals) and sex hormones GWAS (in 189,473 women) in the UK Biobank (UKB) were used in the MR mediation analysis to assess potential mediating roles of these molecules in the causal pathway between NAFLD and PCOS. Replication analysis was conducted using two independent datasets from NAFLD and PCOS GWASs in the UKB and a meta-analysis of data from FinnGen and the Estonian Biobank, respectively. A linkage disequilibrium score regression was conducted to assess genetic correlations between NAFLD, PCOS, glycemic-related traits, and sex hormones using full summary statistics.

**Results:**

Individuals with higher genetic liability to NAFLD were more likely to develop PCOS (OR per one-unit log odds increase in NAFLD: 1.10, 95% CI: 1.02–1.18; *P* = 0.013). Indirect causal effects of NAFLD on PCOS via fasting insulin only (OR: 1.02, 95% CI: 1.01–1.03; *P* = 0.004) and further a suggestive indirect causal effect via fasting insulin in concert with androgen levels were revealed in MR mediation analyses. However, the conditional *F* statistics of NAFLD and fasting insulin were less than 10, suggesting likely weak instrument bias in the MVMR and MR mediation analyses.

**Conclusions:**

Our study suggests that genetically predicted NAFLD was associated with a higher risk of developing PCOS but less evidence for vice versa. Fasting insulin and sex hormones might mediate the link between NAFLD and PCOS.

**Supplementary Information:**

The online version contains supplementary material available at 10.1186/s12916-023-02775-0.

## Background

Polycystic ovary syndrome (PCOS) is the most common cause of anovulatory infertility affecting up to nearly 10% of reproductive-age women [1, 2], and it was recently reported that there are up to ~1.55 million incident cases of women with PCOS globally [3]. In addition, women with PCOS are also at increased risk of developing long-term endocrine complications and cardiometabolic diseases [4]. Linkages between PCOS and non-alcoholic fatty liver disease (NAFLD), which is characterized by excessive hepatic fat accumulation (steatosis) in the absence of significant alcohol consumption [5], have been consistently reported [6, 7], and recent large-scale cohort and meta-analysis studies observed that women with PCOS were associated with a higher risk of NAFLD and its more progressive form, non-alcoholic steatohepatitis [8, 9]. The global prevalence of NAFLD has now reached 32.4%, and its incidence among women has been estimated to be nearly 30 cases per 1000 person-years [10]. The annual burden of PCOS and the direct medical costs of NAFLD and related complications were nearly $8 billion and over $137 billion, respectively, in the USA and Europe [11, 12]. However, to date, there are no effective preventions or therapeutic interventions for the two common and burdensome diseases.

In view of the close connection between these two diseases, recently, a novel hepato-ovarian axis was hypothesized [13]. Moreover, growing evidence showed that insulin resistance and sex hormones (especially increased serum androgen levels) may play essential roles in the pathophysiology of both NAFLD and PCOS [14, 15]. To date, however, the causal relationship between NAFLD and PCOS, and whether there exist potential mediating roles of serum androgen levels and insulin resistance between these two conditions have been insufficiently addressed, because conventional observational analyses are susceptible to residual confounding or reverse causation bias [16].

Mendelian randomization (MR) is a statistical approach that could minimize the risk of bias due to residual confounding or reverse causation as it basically uses germline genetic variants as instrumental variables (IVs) to estimate possible causal effects between modifiable exposures and outcome measures [17].

Thus, in the present study, we investigated the causal relationship between NAFLD and PCOS using a bidirectional two-sample MR analysis. A linkage disequilibrium score regression (LDSR) was then used to assess the genetic correlation between these two diseases. Furthermore, we performed stepwise multivariable MR (MVMR) analyses to test for the mediating roles of glycemic-related traits and serum androgens.

## Methods

### Data sources

**Fig. 1:**
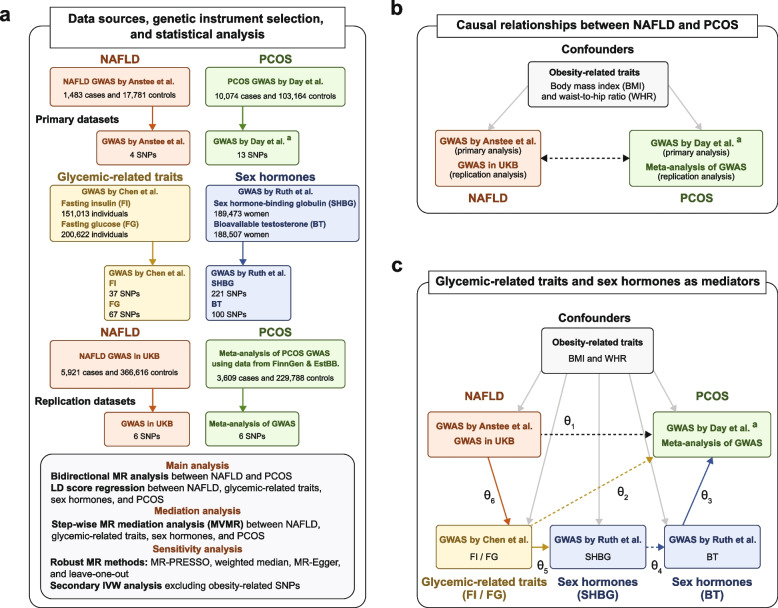
Schematic overview of the study. **a** The solid orange, green, yellow, and blue lines with arrows represent the genetic instrument selection procedure. **b** The dashed black lines with arrows represent bidirectional MR analysis between NAFLD and PCOS. **c** The solid orange, yellow, and blue lines with arrows represent respectively the causal effects of NAFLD on fasting insulin, fasting insulin on sex hormones, and sex hormones on PCOS, which were reported in previous MR studies [18–20]. The dashed yellow and blue lines with arrows represent the causal effects between phenotypes being tested for in this study. The dashed black line with arrows represents the direct causal effect of NAFLD on PCOS. The solid gray lines with arrows represent the causal effects of confounders on NAFLD, PCOS, and mediators [19–21]. a: The full summary statistics of PCOS GWAS by Day et al. included 4890 cases and 20,405 controls (excluding participants in the 23andMe study). *θ*_1_: direct causal effect of NAFLD on PCOS; *θ*_2_: direct causal effect of fasting insulin levels on PCOS; *θ*_3_: direct causal effect of serum bioavailable testosterone (BT) on PCOS; *θ*_4_: direct causal effect of SHBG levels on serum BT; *θ*_5_: direct causal effect of fasting insulin levels on serum SHBG; *θ*_6_: causal effect of NAFLD on fasting insulin; *θ*_2_×*θ*_6_: indirect causal effect of NAFLD on PCOS via fasting insulin levels only; *θ*_3_×θ_4_×*θ*_5_×*θ*_6_: indirect causal effect of NAFLD on PCOS via fasting insulin and sex hormone levels. *Abbreviations*: EstBB, Estonian Biobank; GWAS, genome-wide association study; IVW, inverse-variance weighted; LD, linkage disequilibrium; MR, Mendelian randomization; MVMR, multivariable Mendelian randomization; NAFLD, non-alcoholic fatty liver disease; PCOS, polycystic ovary syndrome; SHBG, sex hormone-binding globulin; SNPs, single nucleotide polymorphisms; UKB, UK Biobank

A schematic overview of the data sources, genetic instrument selection, and statistical analysis in this study is presented in Fig. 1 (panel a). Summary data on NAFLD were obtained from a large genome-wide association study (GWAS) conducted by Anstee et al., which included 1483 cases and 17,781 controls [22]. All NAFLD cases were diagnosed using strict criteria (i.e., liver biopsy). Due to the lack of sex-specific GWAS of NAFLD, we used data from the NAFLD GWAS in the general population and assumed there were no sex-specific genetic effects for NAFLD, as supported by previous studies [23]. Data on PCOS were obtained from a large-scale meta-analysis of PCOS GWAS conducted by Day et al., including 10,074 cases and 103,164 controls of European ancestry, where participants were diagnosed with PCOS according to National Institutes of Health (NIH) criteria, Rotterdam criteria, or self-reported diagnoses [24].

Summary data on glycemic-related traits, including fasting glucose and insulin levels (i.e., a proxy of insulin resistance), were obtained from a GWAS conducted by Chen et al. that involved 200,622 individuals of European ancestry without known diabetes [25]. Summary data on sex hormones were extracted from a GWAS of serum sex hormone-binding globulin (SHBG) and bioavailable testosterone levels (i.e., bioavailable testosterone is calculated using an equation that includes serum total testosterone, SHBG, and albumin concentrations) in up to 189,473 women of European ancestry in the UK Biobank (UKB) [18].

For replication analysis, we used two independent GWASs, a GWAS of NAFLD in the UKB (5921 cases and 366,616 controls) [26] and a GWAS meta-analysis of data on PCOS women (3609 cases and 229,788 controls) in the FinnGen and Estonian Biobank (EstBB), respectively [27]. Information on International Classification of Diseases (ICD) codes that were used to define cases of NAFLD in the UKB and cases of PCOS in the FinnGen and EstBB is presented in Table 1. Detailed information of each GWAS summary statistic in our study can be found in Additional file 1: Table S1.

**Table 1:** The International Classification of Diseases (ICD) code was used to define cases of NAFLD in the UK Biobank and PCOS in the FinnGen and Estonian Biobank

Phenotype	Inclusion criteria	Data source(s)
NAFLD	ICD-10 code K76.0 “Fatty (change of) liver, not elsewhere classified”	UK Biobank
PCOS	ICD-10 code E28.2 “Polycystic ovarian syndrome”	FinnGen; Estonian Biobank
	ICD-9 code 256.4 “Polycystic ovaries”	FinnGen
	ICD-8 code 256.90 “other and unspecified ovarian dysfunction”	FinnGen

### Genetic instrument selection

In the primary MR analysis, ten genome-wide significant (*P* < 5 × 10^−8^) single nucleotide polymorphisms (SNPs) were identified in the biopsy-based NAFLD GWAS [22]. After linkage disequilibrium (LD) clumping (a window of 10Mb and *r*^2^ < 0.001) using the clump-data function in the TwoSampleMR R package [28], 4 bi-allelic SNPs with minor allele frequency (MAF) > 0.01 were retained as genetic instruments (Table 2). Of 14 genome-wide significant SNPs identified in the PCOS GWAS conducted by Day et al. [24], 13 SNPs were selected as genetic instruments for PCOS after excluding rs853854 (MAF close to 0.5) and LD clumping with the same threshold as above.

**Table 2:** SNPs with genome-wide significance were namely used as instrumental variables for NAFLD and PCOS in European ancestry individuals

Phenotype	SNP	CHR	Position	Gene	EA	OA	EAF	Beta	SE	***P*** value	***F*** statistics
Primary analysis datasets											
NAFLD (Anstee et al. GWAS)	rs2068834^a^	2	27839539	*ZNF512*	C	T	0.275	0.264	0.041	8.5×10^−11^	42.1
	rs13118664	4	88239609	*HSD17B13*	T	A	0.251	−0.302	0.053	1.4×10^−08^	32.2
	rs17216588	19	19664077	*CILP2*	T	C	0.077	0.477	0.064	7.2×10^−14^	56.0
	rs738409	22	44324727	*PNPLA3*	G	C	0.224	0.603	0.041	1.5×10^−49^	219.1
PCOS (Day et al. GWAS)	rs7563201	2	43561780	*THADA*	A	G	0.450	−0.108	0.017	3.7×10^−10^	39.5
	rs2178575	2	213391766	*ERBB4*	A	G	0.150	0.166	0.022	3.3×10^−14^	57.7
	rs13164856	5	131813204	*IRF1/RAD50*	T	C	0.730	0.124	0.019	1.4×10^−10^	40.9
	rs804279	8	11623889	*GATA4/NEIL2*	A	T	0.260	0.128	0.018	3.8×10^−12^	48.1
	rs10739076	9	5440589	*PLGRKT*	A	C	0.310	0.110	0.020	2.5×10^−08^	31.0
	rs7864171	9	97723266	*FANCC*	A	G	0.430	−0.093	0.017	2.9×10^−08^	30.8
	rs9696009	9	126619233	*DENND1A*	A	G	0.070	0.202	0.031	8.0×10^−11^	42.2
	rs11031005	11	30226356	*ARL14EP/FSHB*	T	C	0.850	−0.159	0.022	8.7×10^−13^	51.0
	rs11225154	11	102043240	*YAP1*	A	G	0.090	0.179	0.027	5.4×10^−11^	43.2
	rs1784692	11	113949232	*ZBTB16*	T	C	0.820	0.144	0.023	1.9×10^−10^	40.5
	rs2271194	12	56477694	*ERBB3/RAB5B*	A	T	0.420	0.097	0.017	4.6×10^−09^	34.2
	rs1795379	12	75941042	*KRR1*	T	C	0.240	−0.117	0.020	1.8×10^−09^	36.2
	rs8043701	16	52375777	*TOX3*	A	T	0.820	−0.127	0.021	9.6×10^−10^	37.5
Replication analysis datasets											
NAFLD (UKB GWAS)	rs2807834	1	220970593	*MARC1*	T	G	0.309	−0.132	0.021	1.1×10^−10^	41.7
	rs1260326	2	27730940	*GCKR*	T	C	0.388	0.132	0.019	4.0×10^−12^	48.1
	rs17321515	8	126486409	*TRIB1*	G	A	0.473	−0.145	0.019	1.4×10^−14^	59.2
	rs73001065	19	19460541	*MAU2*	C	G	0.068	0.327	0.034	1.7×10^−22^	95.2
	rs429358^a^	19	45411941	*APOE*	C	T	0.154	−0.202	0.027	5.3×10^−14^	56.6
	rs3747207	22	44324855	*PNPLA3*	A	G	0.215	0.340	0.022	3.5×10^−56^	249.4
PCOS (GWAS meta-analysis of data from FinnGen and EstBB)	rs7564590	2	213387900	*ERBB4*	T	C	0.356	0.171	0.026	4.8×10^−11^	43.3
	rs9312937	5	16836005	*MYO10*	T	C	0.554	−0.148	0.026	1.8×10^−08^	31.8
	rs3945628	9	126535553	*DENND1A*	T	C	0.930	−0.340	0.049	2.9×10^−12^	48.7
	rs11031002	11	30215261	*FSHB*	A	T	0.122	0.217	0.038	9.3×10^−09^	32.9
	rs1672716	11	113952497	*ZBTB16*	A	G	0.855	0.206	0.036	9.8×10^−09^	32.8
	rs17880096	22	29105202	*CHEK2*	C	G	0.037	0.335	0.047	1.5×10^−12^	50.0

Genomic positions reported in the GWASs refer to human reference assembly (GRCh37/hg19)

*Abbreviations*: *CHR* chromosome, *EA* effect allele, *EAF* effect allele frequency, *EstBB* Estonian Biobank, *NAFLD* non-alcoholic fatty liver disease, *OA* other alleles (reference allele), *PCOS* polycystic ovary syndrome, *SE* standard error, *SNP* single nucleotide polymorphism

^a^rs2068834 and rs429358 were excluded from the sensitivity analysis due to their genome-wide significant association with obesity

### Proxy variant selection and data harmonization

For genetic instruments that were not available in the outcome GWAS summary data, a proxy variant was looked up (a window of 1 Mb and *r*^2^ ≥ 0.8) in the European 1000 Genomes dataset using the LDlink (https://ldlink.nci.nih.gov/?tab=ldproxy). In the data harmonization procedure, we coded the effect allele and the reference allele in the same strand for both exposure and outcome.

Following the same procedure of LD clumping, proxy variant selection, and data harmonization as above, eligible genetic instruments for glycemic-related traits and serum sex hormone levels are detailed in Additional file 1: Tables S2-S3. In the replication MR analysis, 6 SNPs were selected as genetic instruments for NAFLD and for PCOS, respectively. After getting the eligible IVs, we compared the IV-specific causal effect estimate between the most significant variants used in our analysis (i.e., rs17216588, rs2068834, and rs73001065) and their high LD causal variants, which were previously reported in the literature (i.e., rs58542926 on *TM6SF2* and rs1260326 on *GCKR*) (Additional file 2: Fig. S1).

### Statistical analysis

#### Primary MR analysis

A bidirectional MR analysis was performed to determine the causal relationship between NAFLD and PCOS (Fig. 1, panel b). The random-effects inverse-variance weighted (IVW) method or fixed-effects IVW method was used in the primary MR analysis using the TwoSampleMR R packages [28]. In particular, we used the fixed-effects IVW method when there were three or fewer genetic instruments available; otherwise, the random-effects IVW method was used [29]. To assess the strength of the selected genetic instruments in MR analysis, *F* statistics were calculated, which can be used to examine whether MR estimates are likely to be influenced by weak instrument bias. *F* statistics greater than 10 are generally considered strong [30]. In addition, Cochran’s *Q* test was conducted to assess the heterogeneity of causal effect estimates between NAFLD and PCOS [31].

#### MR mediation analysis

A stepwise MR analysis approach was used to examine whether there exist mediation effects of glycemic-related traits and sex hormones (i.e., serum SHBG and bioavailable testosterone levels) between NAFLD and PCOS (Fig. 1, panel c) [32, 33]. To assess the direct causal effect between NAFLD, glycemic-related traits, sex hormones, and PCOS in each step, we performed an MVMR analysis using the MVMR R package [34]. Conditional *F* statistics were calculated for assessing the strength of the genetic instruments in MVMR analysis (Additional file 1: Tables S4-S6) [35]. Furthermore, to minimize the risk of bias due to horizontal pleiotropy, the MR mediation analysis was conducted after excluding the obesity-related genetic variants which were identified from the PhenoScanner V2 database [36] and the GWAS Catalog [37]. The product of the coefficients method [38] and the multivariate delta method [39] were used to calculate the indirect effects of NAFLD on PCOS via mediators. The detailed stepwise MR mediation analysis and obesity-related SNPs selection procedures can be found in Additional file 1: Table S7 and Additional file 2: “Step-wise MR mediation analysis” and “Obesity-related genetic variants selection.”

#### Replication MR analysis

A replication bidirectional MR analysis between NAFLD and PCOS was performed using two independent NAFLD and PCOS GWAS datasets [26, 27]. To increase the statistical power and precision of our causal estimates, a fixed-effects meta-analysis was conducted to combine the causal estimates derived from the primary MR analysis and the replication MR analysis using the meta R package [40]. We also replicated the findings of the mediation effects of glycemic traits and serum sex hormone levels using the replication analysis datasets.

#### MR sensitivity analysis

To examine the robustness of MR effect estimates to potential invalid genetic variants, we conducted MR-Egger regression [41], weighted median [42], and the Mendelian randomization pleiotropy residual sum and outlier (MR-PRESSO) [43] tests as sensitivity analyses. Unlike the IVW method that assumes all the SNPs are valid IVs [44] when the Instrument Strength must be Independent of the Direct Effect (InSIDE) assumption holds, the MR-Egger regression test could generate a consistent estimate even if all the genetic instruments are invalid [41]. The weighted median model is a robust approach, which could provide consistent estimate results when more than half of the genetic instruments are valid [42]. We used MR-PRESSO to detect the presence of outliers (i.e., potentially pleiotropic SNPs) and estimate the causal effect after excluding outliers [43]. The leave-one-out (LOO) analysis was used to assess whether the causal effect was driven by an influential SNP via recalculating the MR estimates by leaving one instrument out at a time [45]. Moreover, we performed an IVW analysis after excluding obesity-related genetic variants.

#### Genetic correlation analysis

We estimated the genetic correlation between NAFLD, PCOS, glycemic-related traits, and sex hormones via LDSR using the primary and replication GWAS summary datasets, respectively [46].

#### Non-collapsibility of the odds ratio

Non-collapsibility of the odds ratio is a challenge in the mediation analysis when the outcome is binary, such as NAFLD [47]. To assess whether binary outcomes used in MR analysis would impact the estimates and conclusions of our study, a magnetic resonance imaging-derived proton density fat fraction (PDFF) GWAS in the UKB, which was conducted using a linear model [26], was used to replicate the causal associations between NAFLD and PCOS (Additional file 2: “Non-collapsibility of the odds ratio” and Fig. S2).

All statistical analyses were undertaken with R version 4.0.2 (R Foundation for Statistical Computing, Vienna, Austria). Given that up to five risk factors (NAFLD, two glycemic-related traits, and two sex hormone traits) were investigated in MVMR analysis, an estimate with a *P* value, after applying a multiple testing Bonferroni correction, less than 0.01 (*P* = 0.05/5 traits) was considered as strong evidence for causal effects, whereas a *P* value between 0.01 and 0.05 indicated a suggestive causal effect.

## Results

### Causal effect between NAFLD and PCOS

**Fig. 2:**
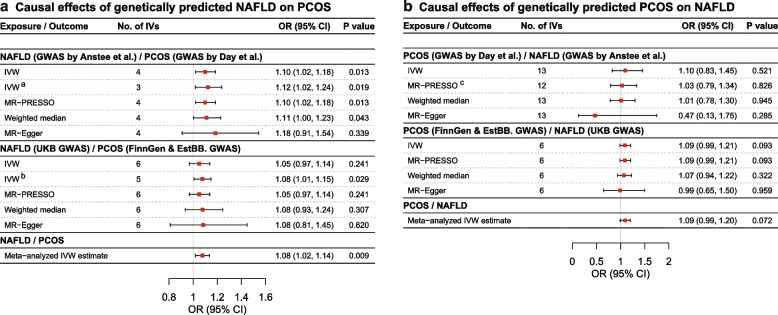
Results of bidirectional MR analysis between NAFLD and PCOS. **a** Causal effect of genetically predicted NAFLD on the risk of PCOS. MR-PRESSO analysis was not applicable to estimate the causal effect of NAFLD on fasting glucose due to the small number of genetic instruments used in the UKB GWAS. **b** Causal effect of genetically predicted PCOS on the risk of NAFLD. The primary MR analysis used data from NAFLD GWAS by Anstee et al. and PCOS GWAS by Day et al. The replication MR analysis used data from the UKB NAFLD GWAS and PCOS GWAS meta-analysis of data from FinnGen and EstBB. A fixed-effects meta-analysis was conducted to combine causal effect estimates derived from the primary and replication MR IVW analysis. a: A secondary IVW analysis was conducted after excluding rs2068834 due to its genome-wide significant association with obesity. b: A secondary IVW analysis was conducted after excluding rs429358 due to its genome-wide significant association with obesity. c: Outlying genetic instruments were excluded in the corrected MR-PRESSO analysis. *Abbreviations*: CI, confidence interval; EstBB, Estonian Biobank; GWAS, genome-wide association study; IVs, instrumental variables; IVW, inverse-variance weighted; NAFLD, non-alcoholic fatty liver disease; OR, odds ratio; PCOS, polycystic ovary syndrome; UKB, UK Biobank

In the primary MR analysis, we found that genetically predicted NAFLD increased the risk of PCOS by 10% (odds ratio [OR] per one-unit log odds increase in NAFLD: 1.10, 95% confidence interval [CI]: 1.02–1.18; *P* = 0.013) (Fig. 2, panel a). Additionally, a total effect equated to an OR for PCOS of 1.12 (95% CI: 1.02–1.24; *P* = 0.019) was estimated in the two-sample MR analysis after excluding an obesity-related SNP (i.e., rs2068834). A similar causal effect (OR: 1.08, 95% CI: 1.01–1.15; *P* = 0.029) was observed in the replication analysis after excluding one obesity-related SNP (i.e., rs429358). Furthermore, the fixed-effects meta-analysis of the IVW causal estimates derived from the primary and replication MR IVW analysis results generated a pooled positive causal effect equated to an OR for PCOS of 1.08 (95% CI: 1.02–1.14; *P* = 0.009) per one-unit log odds increase in NAFLD. In contrast, there was little evidence for a causal effect of genetically predicted PCOS on NAFLD risk, which was consistent with the results of replication analysis and sensitivity analyses (Fig. 2, panel b).

*F* statistics for their respective genetic instruments ranged from 30.8 to 249.4 (Table 2). It suggested that MR analysis results were unlikely to be influenced by weak instrument bias. For the causal effect of NAFLD on the risk of PCOS, Cochran’s *Q* statistics was 1.99 (*P* = 0.575), whereas for the reverse causal effect of POCS on NAFLD risk, Cochran’s *Q* statistics was 29.94 (*P* = 0.003), thereby suggesting a potential heterogeneity across SNP-specific causal effect estimates. The results of the LOO analysis suggested that there was no potentially influential SNP in the primary and replication MR analyses (Additional file 2: Fig. S3). The MR-Egger intercept test results did not show any directional pleiotropy. An outlier-corrected MR-PRESSO test was performed after removing strong outliers among the IVs. Detailed MR-Egger intercept test results and MR-PRESSO global test results can be found in Additional file 1: Tables S8-S9.

Notably, a positive genetic correlation (rg = 0.73, standard error [SE] = 0.27; *P* = 0.007) between NAFLD and PCOS was observed using the primary GWAS summary statistics via LDSR (Additional file 1: Table S10). Although the replication LDSR analysis generated a weaker genetic correlation (rg = 0.27, SE = 0.19; *P* = 0.150), the direction was consistent with that observed in the primary analysis. We further tested pair-wise genetic correlations between all traits in the primary and replication analyses, respectively. Detailed information can be found in Additional file 1: Table S10 and Additional file 2: Fig. S4.

### Causal effects of NAFLD, glycemic-related traits, sex hormones, and PCOS via stepwise MR mediation analysis

**Fig. 3:**
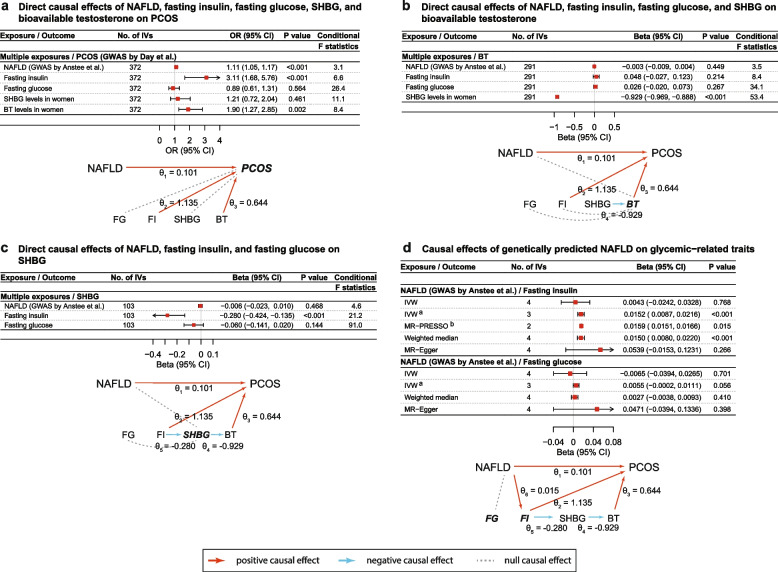
Results of stepwise MR mediation analysis between NAFLD, glycemic-related traits, sex hormones, and PCOS. **a** Direct causal effects of NAFLD, glycemic-related traits, and sex hormones on PCOS. **b** Direct causal effects of NAFLD, glycemic-related traits, and SHBG on serum BT levels. **c** Direct causal effects of NAFLD and glycemic-related traits on serum SHBG levels. **d** Causal effects of NAFLD on glycemic-related traits. MR-PRESSO analysis was not applicable to estimate the causal effect of NAFLD on fasting glucose levels due to the small number of genetic instruments used. *θ*_1_: direct causal effect of NAFLD on PCOS; *θ*_2_: direct causal effect of fasting insulin levels on PCOS; *θ*_3_: direct causal effect of serum BT levels on PCOS; *θ*_4_: direct causal effect of SHBG on serum BT levels; *θ*_5_: direct causal effect of fasting insulin levels on SHBG; *θ*_6_: causal effect of NAFLD on fasting insulin; *θ*_2_×*θ*_6_ indirect causal effect of NAFLD on PCOS via fasting insulin levels only; *θ*_3_×*θ*_4_×*θ*_5_×*θ*_6_: indirect causal effect of NAFLD on PCOS via fasting insulin and sex hormone levels. a: A secondary IVW analysis was conducted after excluding rs2068834 due to its genome-wide significant association with obesity. b: Outlying genetic instruments were excluded in the corrected MR-PRESSO analysis. *Abbreviations*: BT, bioavailable testosterone; CI, confidence interval; FG, fasting glucose; FI, fasting insulin; IVs, instrumental variables; MVMR, multivariable Mendelian randomization; NAFLD, non-alcoholic fatty liver disease; PCOS, polycystic ovary syndrome; SHBG, sex hormone-binding globulin

After excluding obesity-related SNPs, MVMR analysis revealed direct causal effects of NAFLD (OR: 1.11, 95% CI: 1.05–1.17; *P* < 0.001), fasting insulin (OR per increase in natural log-transformed pmol/L fasting insulin: 3.11, 95% CI: 1.68–5.76; *P* < 0.001), and serum bioavailable testosterone levels (OR per increase in natural log-transformed nmol/L bioavailable testosterone: 1.90, 95% CI: 1.27–2.85; *P* = 0.002) on the risk of developing PCOS, respectively (Fig. 3, panel a; Additional file 1: Table S4). By contrast, no causal effect was observed for fasting glucose (OR: 0.89, 95% CI: 0.61–1.31; *P* = 0.564) and SHBG levels (OR: 1.21, 95% CI: 0.72–2.04; *P* = 0.461) on PCOS risk.

In the following steps of the MR mediation analysis, we found strong evidence for a causal effect of serum SHBG levels (beta: −0.929, 95% CI: −0.969 to −0.888; *P* < 0.001) on serum bioavailable testosterone levels (Fig. 3, panel b; Additional file 1: Table S5). Furthermore, an inverse causal association (beta: −0.280, 95% CI: −0.424 to −0.135; *P* < 0.001) between fasting insulin and SHBG levels, whereas a null causal association between either NAFLD (beta: −0.006, 95% CI: −0.023–0.010; *P* = 0.468) or fasting glucose (beta: −0.060, 95% CI: −0.141–0.020; *P* = 0.144) and SHBG levels, was observed (Fig. 3, panel c; Additional file 1: Table S6).

During further estimating causal effects on glycemic-related traits, the MR analysis results did not support any causal effect of genetically predicted NAFLD on fasting insulin levels; nevertheless, a significantly positive causal effect was observed (beta: 0.0152, 95% CI: 0.0087–0.0216; *P* < 0.001) after excluding the pleiotropic obesity-related SNP (Fig. 3, panel d). Meanwhile, little evidence was found to support a causal effect of NAFLD on fasting glucose levels, which was consistent with the results of sensitivity analyses.

Taken together, we found the following two potential mediation pathways between NAFLD and PCOS: (1) an indirect causal effect of NAFLD on PCOS risk via fasting insulin levels only (*θ*_2_×*θ*_6_) (OR: 1.02, 95% CI: 1.01–1.03; *P* = 0.004) and (2) a suggestive indirect causal effect of NAFLD on PCOS risk via circulating levels of fasting insulin, SHBG, and bioavailable testosterone (*θ*_3_×*θ*_4_×*θ*_5_×*θ*_6_) (OR: 1.0025, 95% CI: 1.0002–1.0049; *P* = 0.0323) (Additional file 1: Table S11). These two pathways mediated 14.9% and 2.2% of the total causal effect of NAFLD on PCOS risk, respectively. Detailed estimates of direct and indirect causal effects using the replication datasets can be found in both Additional file 1: Tables S4-S6 and Additional file 2: Fig. S5. The conditional *F* statistics can be found in Fig. 3 (panel a to panel c) and Additional file 1: Table S4-S6, which suggested weak instrument bias may occur in the MVMR analysis for NAFLD and fasting insulin.

## Discussion

In bidirectional MR analyses, we found that genetically predicted NAFLD was causally associated with a higher risk of developing PCOS, whereas there was little evidence for a causal effect of genetically predicted PCOS on the risk of developing NAFLD. In addition, our MR mediation analyses confirmed a direct causal effect of NAFLD on the risk of developing PCOS along with significant indirect causal effects via circulating levels of insulin and sex hormones (namely serum SHBG and bioavailable testosterone levels). Therefore, these findings suggest that fasting insulin and serum androgen levels might play mediating roles in the putative causal pathway, which might be the recently proposed hepato-ovarian axis [13].

Our MR analysis further indicated a causal effect of increased fasting insulin levels (a proxy of insulin resistance) on the risk of PCOS, which was supported by a suggestive causal effect of insulin resistance on PCOS reported in a previous MR study [21]. In the ovarian theca cells, insulin may exert a co-gonadotropin effect on upregulating luteinizing hormone (LH)-induced androgen production [48]. Furthermore, increased serum LH levels and insulin resistance could impair follicle maturation and even cause anovulatory cycles [49]. Previous studies suggested that disruption of insulin receptor signaling in the central nervous system may also contribute to the development of PCOS via the hypothalamic-pituitary-gonadal axis [48–50].

Accumulating evidence supported an association between higher serum androgen levels and PCOS [51]. Moreover, a causal association between increased serum androgen levels and PCOS was confirmed in a recent MR study [18], which was further replicated in the present study. In particular, we found that higher serum bioavailable testosterone levels were causally associated with a higher risk of PCOS, but little evidence was found for a direct causal effect of serum SHBG levels on PCOS risk when adjusting for circulating bioavailable testosterone levels.

A previous MR study reported causal associations between increased fasting insulin and decreased SHBG levels and higher bioavailable testosterone levels, respectively [19]. Our present MR analysis results supported the existence of inverse causal effects of fasting insulin on SHBG levels and SHBG on serum bioavailable testosterone levels; however, there was little evidence for a direct causal effect of fasting insulin levels on serum bioavailable testosterone after adjusting for SBHG levels. Taken together, our findings suggested that higher fasting insulin levels might affect serum bioavailable testosterone levels mainly through serum SHBG reduction.

In our study, obesity was an essential confounder between NAFLD and glycemic-related traits. Previous research reported that obesity could upregulate the pro-inflammatory gene expression, then increase pro-inflammatory cytokine production in the liver, and induce hepatic and systemic insulin resistance [52, 53]. We observed a causal effect of NAFLD on fasting insulin levels, which was consistent with a previous MR study [20], but not fasting glucose levels using genetic instruments for NAFLD excluding one obesity-related SNP (rs2068834). This finding was also supported by studies showing that hepatic steatosis could impair insulin action and then induce insulin resistance in the liver [54]. It is noteworthy that our study did not find any causal associations between genetically predicted NAFLD and serum SHBG or bioavailable testosterone levels after adjusting for obesity and glycemic-related traits, which was inconsistent with observations from some previous studies suggesting that NAFLD patients were more likely to have lower serum SHBG levels [55, 56]. Previous studies found that circulating levels of SHBG could be upregulated by adiponectin, which was inversely associated with obesity [57, 58]. Thus, it is possible to hypothesize that the lower serum SHBG levels and higher bioavailable testosterone levels observed among patients with NAFLD in previous observational studies might be affected by obesity, independent of NAFLD.

Our study has several strengths. First, we used the largest and most recent data from GWASs in European ancestry. Second, we comprehensively tested for the potential mediators in the causal pathway between NAFLD and PCOS. Third, we used independent data sources to validate our causal inference.

There are also some important limitations to this study. First, both the biopsy-based NAFLD GWAS by Anstee et al. [22] and NAFLD GWAS in UKB [26], which were used in the present MR study due to a lack of large-scale sex-specific NAFLD GWAS, were conducted in a sex-combined population. Although previous studies found that NAFLD is a sexual dimorphism condition [59], no sex differences in genetic effects were found for SNPs in genes (or in high LD with genes) including *PNPLA3*, *HSD17B13*, *TM6SF2*, and *GCKR* [23], which were the selected genetic instruments for NAFLD in our MR analyses. Second, the disparity in results of mediation and LDSR analyses between different independent datasets might be, at least in part, attributed to varying definitions used for cases of NAFLD and PCOS. In the datasets for primary analysis, cases of NAFLD and PCOS were diagnosed using strict criteria (i.e., liver biopsy and NIH or Rotterdam criteria, respectively), whereas cases of both conditions were identified only by ICD codes in the datasets for replication analysis. PCOS was identified in the FinnGen study using electronic health records since 1968, which may not be as accurate as data using the recent diagnostic criteria. Third, due to lacking independent large-scale glycemic-related traits and sex hormones GWAS, sample overlap exists between fasting insulin and fasting glucose and between SHBG and bioavailable testosterone levels in the mediation analysis. However, we tried our best to search for all the available GWASs and selected independent NAFLD and PCOS GWASs in primary and replication analyses, respectively. Therefore, our main causal effect estimates between NAFLD and PCOS were unlikely to be affected by sample overlap. Fourth, although each exposure was strongly predicted by the genetic variants in the two-sample MR analysis, the MVMR analysis was still likely to be biased by the conditional weak instruments [34]. And the weak instrument bias cannot be ruled out in both primary and replication MR mediation analyses. The underlying mechanisms of the suggestive causal pathways between NAFLD and PCOS in our study need further investigation.

It is noteworthy that the primary MR analysis found a positive causal effect of NAFLD on PCOS risk using the NAFLD GWAS by Anstee et al., which was conducted within the population of South Europe [22], while NAFLD and PCOS GWASs used in the replication analysis were conducted in West and North Europe (i.e., the UK and Finland/Estonia) [26, 27]. Although a statistically non-significant causal effect of NAFLD on PCOS risk was observed in our replication MR analysis, the causal effect magnitude and direction were consistent with the primary analysis results. Moreover, the statistically significant pooled MR estimates of primary and replication analysis results supported a causal effect of NAFLD on PCOS risk. Thus, collectively, our results can largely be generalized to European populations.

## Conclusions

Our study supported a causal association between genetically predicted NAFLD and higher risk of developing PCOS. Moreover, the underlying mechanisms from NAFLD to PCOS might be linked via higher circulating levels of fasting insulin (a proxy of insulin resistance) and sex hormones (mainly bioavailable testosterone levels). The findings of this study suggested the potential clinical and public health significance of early diagnosis and management of NAFLD for future PCOS prevention. Given that the likelihood of our MVMR analysis results being potentially biased by conditional weak instruments cannot be ruled out, the mediating biomarkers investigated in this study should be cautiously considered as potential therapeutic targets and need to be validated in future larger genetic studies and intervention studies.

## Supplementary Information

**Additional file 1: Table S1.** Key characteristics of participating studies. **Table S2.** GWAS significant SNPs used as genetic instruments for fasting insulin and fasting glucose. **Table S3.** GWAS significant SNPs used as genetic instruments for serum SHBG levels and bioavailable testosterone levels in women. **Table S4.** Direct causal effects of NAFLD, fasting insulin, fasting glucose, serum SHBG levels, and serum bioavailable testosterone levels on PCOS risk via multivariable MR analysis. **Table S5.** Direct causal effects of NAFLD, fasting insulin, fasting glucose, and serum SHBG levels on serum bioavailable testosterone levels via multivariable MR analysis. **Table S6.** Direct causal effects of NAFLD, fasting insulin, and fasting glucose on serum SHBG levels via multivariable MR analysis. **Table S7.** Obesity-related genome-wide significant genetic variants. **Table S8.** Directional pleiotropy test using MR-Egger intercepts. **Table S9.** Horizontal pleiotropy test using MR-PRESSO. **Table S10.** Linkage disequilibrium score regression results on genetic correlations between NAFLD, fasting insulin, fasting glucose, SHBG, BT, and PCOS. **Table S11.** Indirect causal effects between NAFLD and PCOS via fasting insulin, serum SHBG levels, and serum bioavailable testosterone levels through step-wise MR analysis.

**Additional file 2: **Additional information on data sources. Additional methods and results. **Fig. S1.** Causal effects of NAFLD on PCOS using specific variants. **Fig. S2.** Results of bidirectional MR analysis between NAFLD and PCOS using linear PDFF GWAS and binary NAFLD GWAS. **Fig. S3.** Leave-one-out analysis results of MR causal effects between NAFLD and PCOS. **Fig. S4.** Genetic correlations between NAFLD, FI, FG, SHBG, BT, and PCOS in LDSR analysis. **Fig. S5.** Results of step-wise MR mediation analysis for causal associations between NAFLD, glycemic-related traits, sex hormones, and PCOS using replication GWAS datasets. **Fig. S6.** The overview of the step-wise MR mediation analysis between NAFLD and PCOS via glycemic-related traits and sex hormones. **Fig. S7.** A schematic diagram of calculating the indirect causal effect of NAFLD on PCOS via fasting insulin and sex hormones. **Fig. S8.** Causal effect of NAFLD on PCOS using two-sample MR after excluding BMI-related IV and using MVMR.

## Data Availability

All data generated or analyzed during this study are included in Additional file 2: “Additional information on data sources.”

## Abbreviations

BT: Bioavailable testosterone
CHR: Chromosome
CI: Confidence interval
EA: Effect allele
EAF: Effect allele frequency
EstBB: Estonian Biobank
FG: Fasting glucose
FI: Fasting insulin
GWAS: Genome-wide association study
ICD: International Classification of Diseases
IVs: Instrumental variables
IVW: Inverse-variance weighted
LD: Linkage disequilibrium
LDSR: Linkage disequilibrium score regression
LH: Luteinizing hormone
LOO: Leave-one-out
MR: Mendelian randomization
MR-PRESSO: Mendelian randomization pleiotropy residual sum and outlier
MVMR: Multivariable Mendelian randomization
NAFLD: Non-alcoholic fatty liver disease
NIH: National Institutes of Health
OA: Other alleles (reference allele)
OR: Odds ratio
PCOS: Polycystic ovary syndrome
SE: Standard error
SHBG: Sex hormone-binding globulin
SNPs: Single nucleotide polymorphisms
UKB: UK Biobank
