# Supplementary material for: The hepato-ovarian axis: genetic evidence for a causal association between non-alcoholic fatty liver disease and polycystic ovary syndrome
Source: BMC Med. 2023 Feb 20;21:62. doi: 10.1186/s12916-023-02775-0 (PMC9940436; doi:10.1186/s12916-023-02775-0)
Supplement: Supplementary file 2 — Additional file 2: Additional information on data sources. Additional methods and results. Fig. S1. Causal effects of NAFLD on PCOS using specific variants. Fig. S2. Results of bidirectional MR analysis between NAFLD and PCOS using linear PDFF GWAS and binary NAFLD GWAS. Fig. S3. Leave-one-out analysis results of MR causal effects between NAFLD and PCOS. Fig. S4. Genetic correlations between NAFLD, FI, FG, SHBG, BT, and PCOS in LDSR analysis. Fig. S5. Results of step-wise MR mediation analysis for causal associations between NAFLD, glycemic-related traits, sex hormones, and PCOS using replication GWAS datasets. Fig. S6. The overview of the step-wise MR mediation analysis between NAFLD and PCOS via glycemic-related traits and sex hormones. Fig. S7. A schematic diagram of calculating the indirect causal effect of NAFLD on PCOS via fasting insulin and sex hormones. Fig. S8. Causal effect of NAFLD on PCOS using two-sample MR after excluding BMI-related IV and using MVMR. [file 12916_2023_2775_MOESM2_ESM.docx]

**Additional file 2**

[**Additional information on data sources** 2](#_Toc125149537)

[**Additional methods and results** 4](#_Toc125149538)

[**Fig S1. Causal effects of NAFLD on PCOS using specific variants.** 8](#_Toc125149539)

[**Fig S2. Results of bidirectional MR analysis between NAFLD and PCOS using linear PDFF GWAS and binary NAFLD GWAS.** 9](#_Toc125149540)

[**Fig S3. Leave-one-out analysis results of MR causal effects between NAFLD and PCOS.** 10](#_Toc125149541)

[**Fig S4. Genetic correlations between NAFLD, FI, FG, SHBG, BT, and PCOS in LDSR analysis.** 11](#_Toc125149542)

[**Fig S5. Results of step-wise MR mediation analysis for causal associations between NAFLD, glycemic-related traits, sex hormones, and PCOS using replication GWAS datasets.** 12](#_Toc125149543)

[**Fig S6. The overview of the step-wise MR mediation analysis between NAFLD and PCOS via glycemic-related traits and sex hormones.** 13](#_Toc125149544)

[**Fig S7. A schematic diagram of calculating the indirect causal effect of NAFLD on PCOS via fasting insulin and sex hormones.** 14](#_Toc125149545)

[**Fig S8. Causal effect of NAFLD on PCOS using two-sample MR after excluding BMI-related IV and using MVMR.** 15](#_Toc125149546)

# **Additional information on data sources**

**Non-alcoholic fatty liver disease (NAFLD) genome-wide association study (GWAS) conducted by Anstee et al.**

The NAFLD GWAS conducted by Anstee et al. included 1,483 NAFLD cases (average age is 50.1 years old; 47.3% are women) from 10 European tertiary liver centers (Freeman Hospital, Newcastle upon Tyne Hospitals NHS Trust, Newcastle upon Tyne, UK; Addenbrooke's Hospital, Cambridge, UK; Nottingham University Hospitals NHS Trust, Nottingham, UK; Inselspital Hospital, Bern, Switzerland; Antwerp University Hospital, Belgium; Pitié-Salpêtrière Hospital, Paris, France; University Hospital, Linköping, Sweden & Oskarshamn County Hospital, Sweden; University Medical Center, Mainz, Germany; Città della Salute e della Scienza di Torino Hospital, Turin, Italy; Section of Gastroenterology and Hepatology, University of Palermo, Italy; and Foundation IRCCS Ca' Granda Ospedale Maggiore Policlinico, Milan, Italy), and the 17,781 controls were chosen from the general population with genome-wide genotype data including [Wellcome Trust Case Control Consortium (<https://www.wtccc.org.uk/>), Hypergenes cohort (<http://www.hypergenes.eu/dissemination.html#pub>), KORA (<https://epi.helmholtz-muenchen.de/>), and Understanding Societies (<https://www.understandingsociety.ac.uk/>)] (Additional file 1: **Table S1**). The GWAS adjusted for sex and the first five principal components. Genomic positions reported in the GWAS of NAFLD conducted by Anstee et al. refer to human reference assembly (GRCh37/hg19). Summary statistics were downloaded from the NHGRI-EBI GWAS Catalog (<https://www.ebi.ac.uk/gwas/>) for study GCST90011885 (Anstee et al., 2020) on 08/10/2021.

**NAFLD GWAS in the UK Biobank (UKB)**

A total of 372,537 European ancestry individuals (5,921 NAFLD cases and 366,616 controls) aged between 40 and 69 years at recruitment were included in the GWAS. Patients with NAFLD were defined using the International Classification of Diseases, Tenth Revision (ICD-10) K76.0 "Fatty (change of) liver, not elsewhere classified" (Additional file 1: **Table S1**). The UKB NAFLD GWAS adjusted for sex, age and the first 20 principal components. Genomic positions reported in the UKB GWAS of NAFLD refer to human reference assembly (GRCh38/hg38). Summary statistics were downloaded from <https://www.decode.com/summarydata>.

**Polycystic ovary syndrome (PCOS) GWAS conducted by Day et al.**

Patients with PCOS were diagnosed according to the National Institutes of Health (NIH), Rotterdam criteria, or self-reported diagnoses (23andMe). The final PCOS GWAS full summary statistics included 4,890 cases and 20,405 controls, after excluding participants in 23andMe (Additional file 1: **Table S1**). The GWAS adjusted for age. Genomic positions reported in the PCOS GWAS conducted by Day et al. refer to human reference assembly (GRCh37/hg19). Summary statistics were downloaded from <https://www.repository.cam.ac.uk/handle/1810/283491>.

Seven cohorts were included in this PCOS GWAS: 1) The Rotterdam study included 1,184 PCOS cases and 5,799 population-based Rotterdam study controls. NIH and Rotterdam criteria were used to diagnose PCOS; 2) the UK PCOS study included 670 PCOS cases and 1,379 controls. Both NIH and Rotterdam criteria were used to diagnose PCOS. Controls were selected from the 1958 British Birth Cohort; 3) the Estonian Genome Center of the University of Tartu (EGCUT) cohort (<http://www.biomarcare.eu/the-cohorts-biomarcare/24-population-based-cohorts/34-estonian-genome-center-of-the-university-of-tartu-egcut>) included 157 cases and 2,807 population-based controls. Rotterdam criteria were used to diagnose PCOS; 4) the deCODE cohort (<https://www.decode.com/>) included 658 cases and 6,774 population-based controls. Both NIH and Rotterdam criteria were used to diagnose PCOS; 5) the Chicago PCOS cohort (NUgene Project [<https://www.cgm.northwestern.edu/cores/nugene/index.html>] and University of Chicago Clinical Research Center, IL, USA) included 984 cases and 2,963 population-based controls. NIH criteria were used to diagnose PCOS; 6) the Boston PCOS cohort (Massachusetts General Hospital, Boston, USA) included 485 cases and 407 controls. NIH criteria were used to diagnose PCOS; and 7) the 23andMe, Inc. (Mountain View, CA, USA) cohort included 5,184 cases and 82,759 controls. Women diagnosed with PCOS (self-reported diagnosis) were defined as PCOS cases. In addition, 14 top hits which identified in the PCOS GWAS meta-analysis by Day et al. have consistent directions across studies using different diagnosis criteria, except for one variant (rs804279) with significant heterogeneity across different groups (*P* = 2.67 × 10^-5^, I^2^ = 0.91).

**Meta-analysis of PCOS GWAS using data from FinnGen and Estonian Biobank (EstBB)**

In the FinnGen PCOS GWAS (data freeze release 6), women with PCOS were identified according to the ICD-10 code E28.2 "Polycystic ovarian syndrome", ICD-9 code 256.4 "Polycystic ovaries", or ICD-8 code 256.90 "Other and unspecified ovarian dysfunction". In the EstBB PCOS GWAS, women with PCOS were identified according to the ICD-10 code E28.2. Women without PCOS were defined as controls. The PCOS GWAS meta-analysis adjusted for age and the first ten principal components. Genomic positions reported in the PCOS GWAS meta-analysis of data from the FinnGen and EstBB refer to human reference assembly (GRCh37/hg19). Summary statistics were downloaded from the NHGRI-EBI GWAS Catalog for study GCST90044902 (Tyrmi et al., 2021) on 05/03/2022.

**Glycemic-related traits GWAS**

The glycemic-related traits GWASs included up to 131 cohorts and 281,416 individuals (Additional file 1: **Table S1**). The blood fasting insulin and glucose levels were namely measured in pmol/L and mmol/L, where fasting insulin was natural log-transformed. The detailed information on each participant study can be found in “Supplementary Table 1” in the original paper. The fasting insulin and fasting glucose GWASs adjusted for body mass index (BMI), study-specific covariates, and principal components. Genomic positions reported in the GWAS of glycemic-related traits conducted by Chen et al. refer to human reference assembly (GRCh37/hg19). Summary statistics were downloaded from the Meta-Analyses of Glucose and Insulin-related traits Consortium (MAGIC) ([www.magicinvestigators.org](http://www.magicinvestigators.org)).

**Sex hormones GWAS**

The sex hormones GWASs in the UKB conducted by Ruth et al. included 189,473 women for serum sex hormone-binding globulin (SHBG) levels in women and 188,507 women for bioavailable testosterone (BT) levels in women (Additional file 1: **Table S1**). The serum SHBG and bioavailable testosterone levels were measured in nmol/L, where SHBG was rank-based inverse normal transformed and bioavailable testosterone was natural log-transformed, respectively. Women’s SHBG levels and bioavailable testosterone levels GWASs unadjusted for BMI were used in the study. Genomic positions reported in the GWAS of sex hormones conducted by Ruth et al. refer to human reference assembly (GRCh37/hg19). Summary statistics were downloaded from the NHGRI-EBI GWAS Catalog for study GCST90012102 and GCST90012107 (Ruth et al., 2020) on 11/28/2021.

**Sample overlap**

The NAFLD and PCOS GWASs for primary and replication analyses were conducted in independent populations. The main findings of bidirectional two-sample MR analysis were unlikely to be impacted by sample overlap. However, summary data on fasting insulin and glucose were extracted from the same GWAS. We used the latest large-scale glycemic-related trait GWAS conducted by the MAGIC consortium (<https://magicinvestigators.org/downloads>). To our best knowledge, the MAGIC consortium provided the largest glycemic-related trait GWASs of European ancestry. We were not able to identify another independent large-scale GWAS, which has a similar sample size, for glycemic traits. And the sex hormone level GWAS in UKB conducted by Ruth et al. was the only large-scale GWAS that we can identify and it has been increasingly used in recent MR studies. Moreover, there is a sample overlap between sex hormones GWAS and UKB NAFLD GWAS in the replication analysis.

# **Additional methods and results**

**Instruments selection-genetic variants and well-known variants**

As suggested by the reviewer, we compared the causal effect estimates between genetic variants used in our analysis and well-known variants reported in previous genetic studies (indeed they were in linkage disequilibrium [LD]). A high level of concordance between causal effect estimates was observed (Additional file 2: **Fig. S1**).

**Using MVMR and two-step MR in mediation analysis**

In the MVMR analysis, we used the IVs which were associated with more than one risk factor to estimate the direct causal effect of main exposure after controlling for other risk factors, even if there are causal relationships between risk factors. In the two-step MR analysis, the genetic instruments for the exposure of interest and mediators were selected for estimating the causal effect of the exposure on the mediator and the mediator on the outcome, respectively. After that, an indirect effect of the exposure on the outcome via mediators was calculated based on the above estimated causal effects. The direct and indirect causal effects estimated by MVMR have been increasingly used in the two-step MR analysis for mediation analysis.

**Non-collapsibility of the odds ratio**

To evaluate whether the non-collapsibility of the odds ratio impacted the estimates and conclusions in our MR analysis, we conducted a sensitivity analysis by repeating the main analysis using two different types of NAFLD outcomes; one was extracted from magnetic resonance imaging-derived proton density fat fraction (PDFF) GWAS conducted using a liner regression model in 36,116 Britons of European ancestry from the UKB, and the other was conducted using a logistic regression model in 5,921 cases, where NAFLD cases were diagnosed according to the ICD 10 code K76.0, and 366,616 controls. Bidirectional MR analysis was conducted to test the concordance of the causal effect estimates between two different types of NAFLD outcomes (i.e., continuous and dichotomous).

In Additional file 2: **Fig. S2** (panel a), we tested for the causal effects of NAFLD and PDFF on PCOS (meta-analysis PCOS GWAS using data from GWAS by Day et al. and GWAS in FinnGen and EstBB), respectively. The results showed a consistent direction and effect size (OR_NAFLD_ per unit log odds increase in NAFLD: 1.06, 95% CI: 0.99 to 1.14, *P* = 0.11; OR_PDFF_ per SD increase in PDFF: 1.08, 95% CI: 0.98 to 1.19, *P* = 0.11). Meanwhile, we also tested the causal effects of PCOS on NAFLD and PDFF (Additional file 2: **Fig. S2**, panel b and panel c**)**. In panel b and panel c, we tested for the causal effects of PCOS on NAFLD and PDFF, respectively. Comparing the causal associations between panel b and panel c, we observed little evidence to support a causal effect of PCOS (measured in FinnGen & EstBB) on NAFLD (OR: 1.09, 95% CI: 0.99 to 1.21, *P* = 0.09) and PDFF (beta: 0.03, 95% CI: -0.0008 to 0.07, *P* = 0.06) and PCOS (reported by Day et al.) on NAFLD (OR: 0.98, 95% CI: 0.87 to 1.09, *P* = 0.70) and PDFF (beta: 0.02, 95% CI: -0.03 to 0.07, *P* = 0.38). The causal effect directions were generally consistent across comparisons between using binary NAFLD and continuous PDFF outcomes. In addition, to our best knowledge, there have been no available continuous measurements for PCOS GWAS in European ancestry, therefore we were not able to conduct a similar sensitivity analysis with regard to PCOS. Taken together, we believe that there is little influence of using binary GWAS summary statistics on our MR estimates and conclusions.

**Step-wise MR mediation analysis**

Previous studies found causal associations between NAFLD, PCOS, obesity, insulin resistance, and sex hormones; however, the causal pathways from NAFLD to PCOS via mediators have yet to be established. In the present study, we first conducted an MVMR analysis including NAFLD, fasting insulin, fasting glucose, SHBG, and bioavailable testosterone as exposures to estimate the direct causal effects of each exposure on the risk of PCOS (Additional file 2: **Fig. S6**, panel a). After that, a similar procedure was conducted to test the direct effects of multiple exposures on serum bioavailable testosterone and SHBG levels, respectively (Additional file 2: **Fig. S6**, panel b and panel c). Then, a two-sample MR analysis was used to estimate the causal effects of NAFLD on glycemic-related traits (Additional file 2: **Fig. S6**, panel d). Taken together, the causal effects between NAFLD, PCOS, and each mediator, and the potential mediation pathways between NAFLD and PCOS were established (Additional file 2: **Fig. S6**, panel e). All the MVMR and two-sample MR analyses were conducted including or excluding the obesity-related single nucleotide polymorphisms (SNPs), respectively (Additional file 1: **Tables S4-S6**).

After getting the direct causal relationships between each trait, we followed the method of two-step MR and multiple mediators to estimate the indirect causal effects of NAFLD on PCOS via potential mediators (i.e., glycemic-related traits and sex hormones). The product of the coefficients method and the multivariate delta method were used to calculate the indirect effects of NAFLD on PCOS via mediators. Assuming the effect size of the mediator on the outcome is $\theta_{1}$ with the standard error $\sigma_{1}$, and the effect size of the exposure on the mediator is $\theta_{2}$ with the standard error $\sigma_{2}$, the indirect effect of the exposure on the outcome would be calculated as $\theta_{3}=\theta_{1}\times\theta_{2}$ with the standard error $\sigma_{3}=\sqrt{\theta_{1}^{2}\sigma_{2}^{2}+\theta_{2}^{2}\sigma_{1}^{2}}$.

To generate the indirect causal effect of NAFLD on PCOS via fasting insulin and sex hormones, we first calculated the indirect causal effect of SHBG on PCOS via bioavailable testosterone using the direct causal effect of SHBG on bioavailable testosterone and the direct causal effect of bioavailable testosterone on PCOS (Additional file 2: **Fig. S7**, panel a), which we tested in the MVMR analysis. Then we used the direct causal effect of fasting insulin on SHBG and the indirect causal effect of SHBG on PCOS to estimate the indirect causal effect of fasting insulin on PCOS (Additional file 2: **Fig. S7**, panel b). Finally, the indirect effect of NAFLD on PCOS via fasting insulin, SHBG, and BT were calculated using the direct causal effect of NAFLD on fasting insulin and the indirect causal effect of fasting insulin on PCOS following the same procedure (Additional file 2: **Fig. S7**, panel c).

**Obesity-related genetic variants selection**

Previous MR studies reported that obesity is a common causal risk factor for NAFLD, PCOS, and potential mediators used in the present study. Thus, to minimize the risk of bias due to potentially violating the IV-3 assumption (i.e., exclusion restriction assumption, that is, genetic variants can only affect the outcome via the exposure of interest other than alternative pathways), we excluded obesity-related genetic variants, which were identified from the published GWASs, in the MR sensitivity analysis. Keywords, including "Body mass index", "Waist hip ratio", and "Waist to hip ratio", were used for genetic variants look-up in the PhenoScanner V2 database (<http://www.phenoscanner.medschl.cam.ac.uk/>, European ancestry, *P* < 5 × 10^-8^, r^2^ = 0.8). In addition, all the genetic instruments (or SNPs in high linkage disequilibrium with the genetic instruments) associated with "body mass index" or "waist-hip ratio" were identified in the GWAS Catalog (<https://www.ebi.ac.uk/gwas/>). As a result, genetic variants (rs2068834 in NAFLD GWAS by Anstee et al.; rs429358 in replication NAFLD GWAS) were excluded from MR sensitivity analyses since it was genome-wide significantly associated with obesity (**Table 2**). No obesity-related genetic variants were excluded from glycemic-related traits GWAS summary data since the original GWAS had already adjusted for BMI. Characteristics of all the obesity-related SNPs can be found in Additional file 1: **Table S7**.

**Comparing excluding BMI-related IV and including BMI in MVMR analysis**

As per one of the referees’ kind suggestions, we agree that including BMI or waist-to-hip ratio (WHR) in the MVMR analysis would be another option to control for obesity. However, considering that we have already included NAFLD, FI, FG, SHBG, and BT in the MVMR, if we additionally included BMI and WHR, the increased number of genetic variants in MVMR would dramatically decrease the statistical power and strength of IVs. More importantly, based on previous studies, we believed that obesity is a common cause of NAFLD and PCOS rather than a mediator. In our study, one of the main aims was to investigate the potential mediating roles of glycemic traits and sex hormones in the causal pathway between NAFLD and PCOS. For these reasons, we decided to include glycemic traits and sex hormones in the MVMR while excluding obesity-related genetic variants from the IV set.

To further evaluate the impact of excluding BMI-related SNPs or including BMI in the MVMR, we compared the estimated causal effects of NAFLD on PCOS between excluding one BMI-related SNP (i.e., rs2068834) (Additional file 2: **Fig. S8**, panel a) and controlling for BMI in the MVMR (Additional file 2: **Fig. S8**, panel b). The primary NAFLD GWAS by Anstee et al., PCOS GWAS by Day et al., and the 2018 GIANT and UKB meta-analysis BMI GWAS (Zenodo, <https://doi.org/10.5281/zenodo.1251813>), which included up to 484,680 individuals, were used in two-sample MR and MVMR, respectively. We observed consistent causal effect estimates between the two different analysis strategies: the causal effect of NAFLD on PCOS after excluding BMI-related SNP (i.e., rs2068834) (OR: 1.12, 95% CI: 1.02 to 1.24, *P* = 0.019) vs that (OR: 1.08, 95% CI: 1.02 to 1.14, *P* = 0.014) generated in MVMR analysis after controlling for BMI, suggesting the robustness of results estimated in our MR analysis.

**Conditional F-statistics**

We calculated the conditional F statistics for instruments strength (Additional file 1: **Tables S4-S6**) using the MVMR R package. Although the conventional F statistic for each IV was tested (Table S2-S3) and no weak instrument was observed, the conditional F statistics for MVMR indicated the weak (conditional F statistics < 10) IV strength for NAFLD and fasting insulin in the primary MVMR analysis. After LD clumping, only 4 eligible IVs remained for primary MR analysis. However, there were more than 200 IVs for SHBG and 100 IVs for bioavailable testosterone. The conditional F statistics for NAFLD were still less than 10 in the MVMR analysis with different exposures, including fasting insulin, fasting glucose, and sex hormones (Additional file 1: **Tables S4-S6**). To maintain relatively optional conditional F statistics, we limited the number of potential mediators in the MVMR. Obesity-related SNPs were excluded from the IV sets rather than including obesity in the MVMR analysis. In addition, we replicated the MVMR analysis using another large-scale independent NAFLD GWAS in 5,921 cases and 366,616 controls, but only 6 eligible genetic variants were identified in this GWAS. The weak strength of instruments strength was observed in the replication MVMR as well (Additional file 1: **Tables S4-S6**). Considering the weak instruments used in MVMR, we need to interpret the findings and the suggestive indirect causal pathways of NAFLD on PCOS via fasting insulin and sex hormones with caution. The estimates of the causal effects in MVMR and two-step MR could be biased due to weak instruments, especially for a binary outcome (such as PCOS).


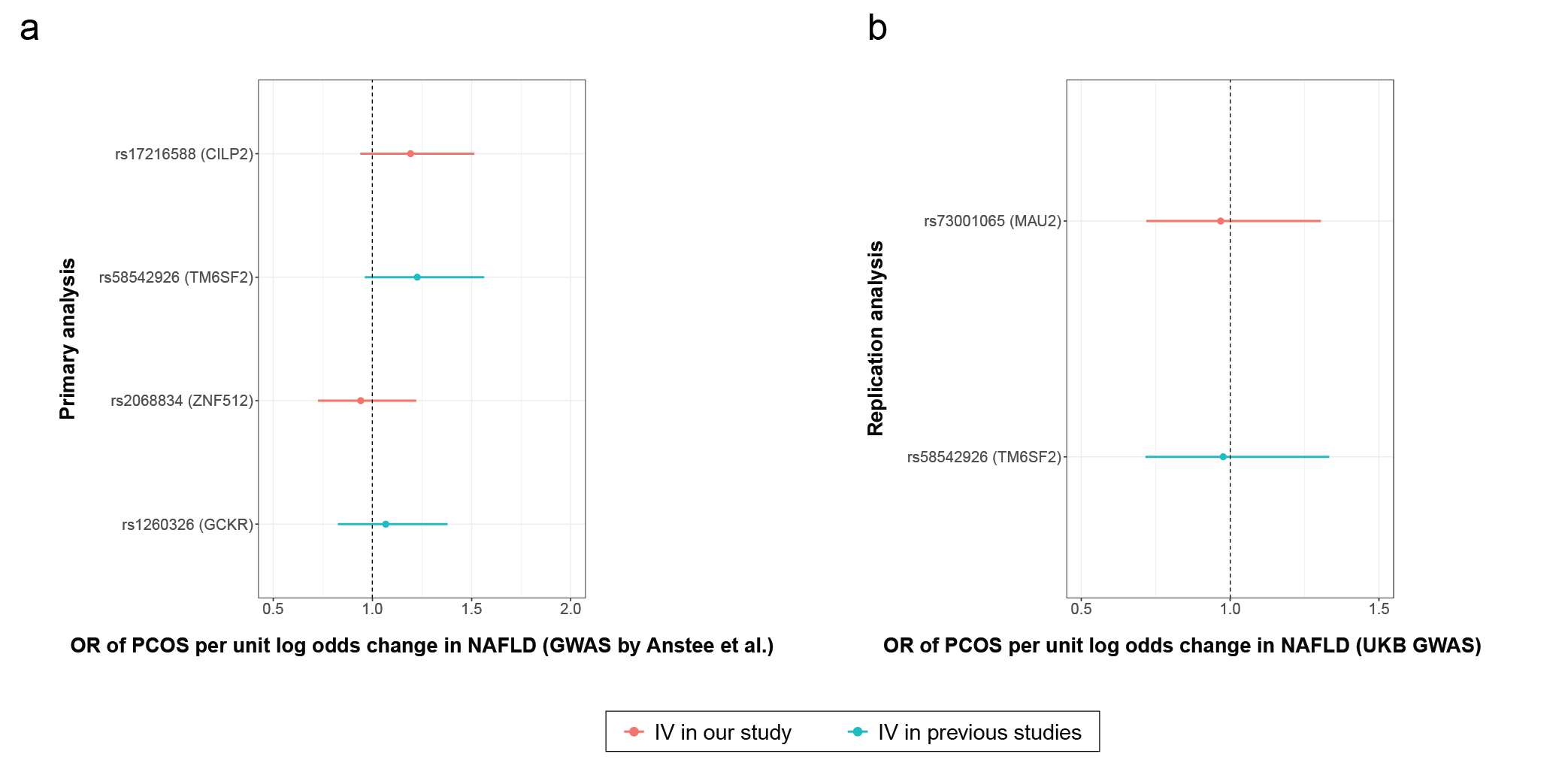


# **Fig S1. Causal effects of NAFLD on PCOS using specific variants.**

Panel a. SNP specific causal effects of NAFLD on PCOS in the primary analysis. Panel b. SNP specific causal effects of NAFLD on PCOS in the replication analysis.

*The LD information for each pair: rs17216588 and rs58542926 (R2 = 0.84, D’ = 0.95), rs2068834 and rs1260326 (R2 = 0.36, D’ = 0.81), and rs73001065 and rs58542926 (R2 = 0.88, D’ = 0.97).

Abbreviations: GWAS, genome-wide association study; IV, instrumental variable; NAFLD, non-alcoholic fatty liver disease; OR, odds ratio; PCOS, polycystic ovary syndrome.


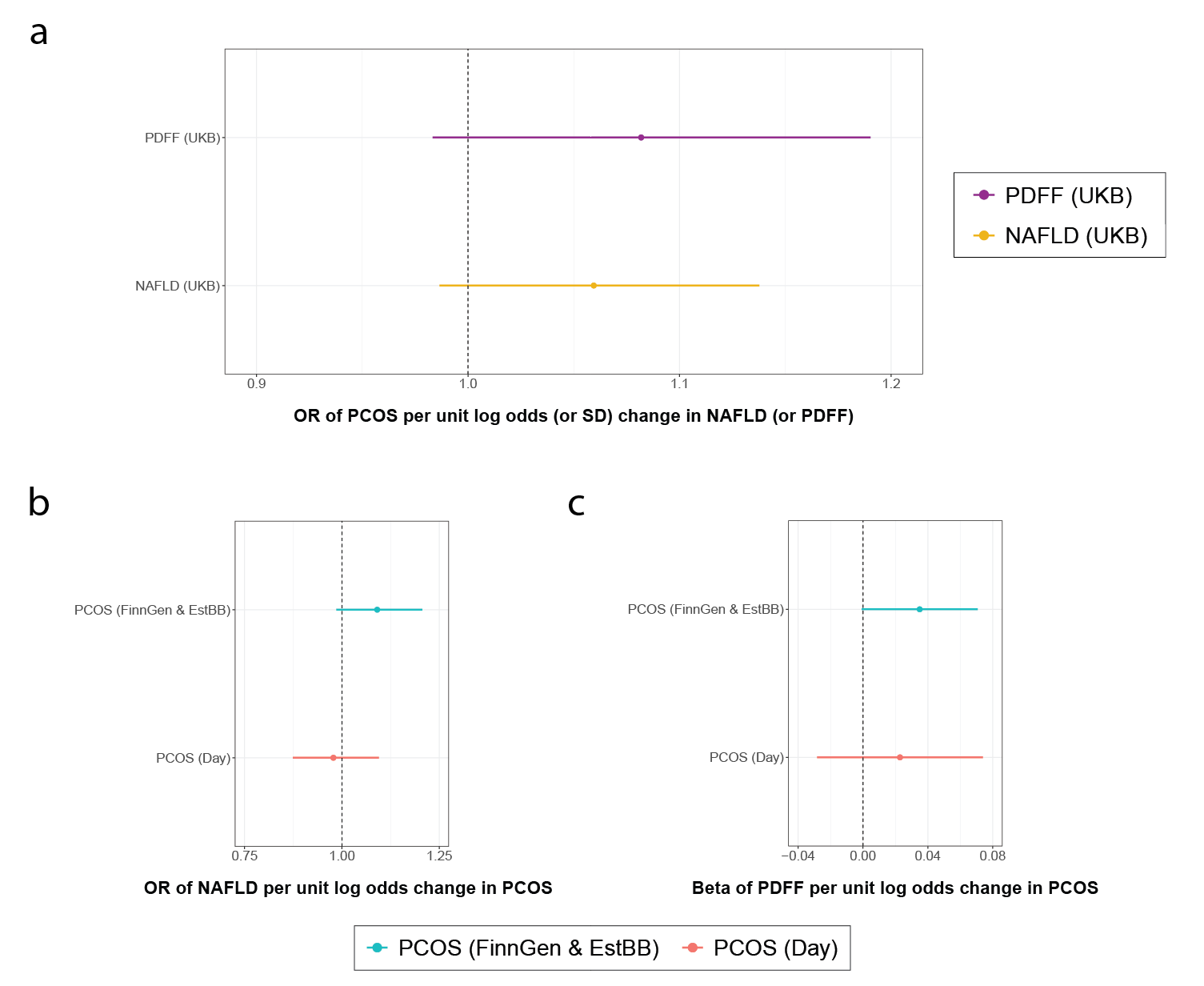


# **Fig S2. Results of bidirectional MR analysis between NAFLD and PCOS using linear PDFF GWAS and binary NAFLD GWAS.**

Panel a. Causal effects of PDFF and NAFLD on PCOS. Panel b. Causal effects of PCOS on NAFLD. Panel c. Causal effects of PCOS on PDFF.

Abbreviations: NAFLD, non-alcoholic fatty liver disease; PCOS, polycystic ovary syndrome; PDFF, proton density fat fraction; SD, standard deviation.


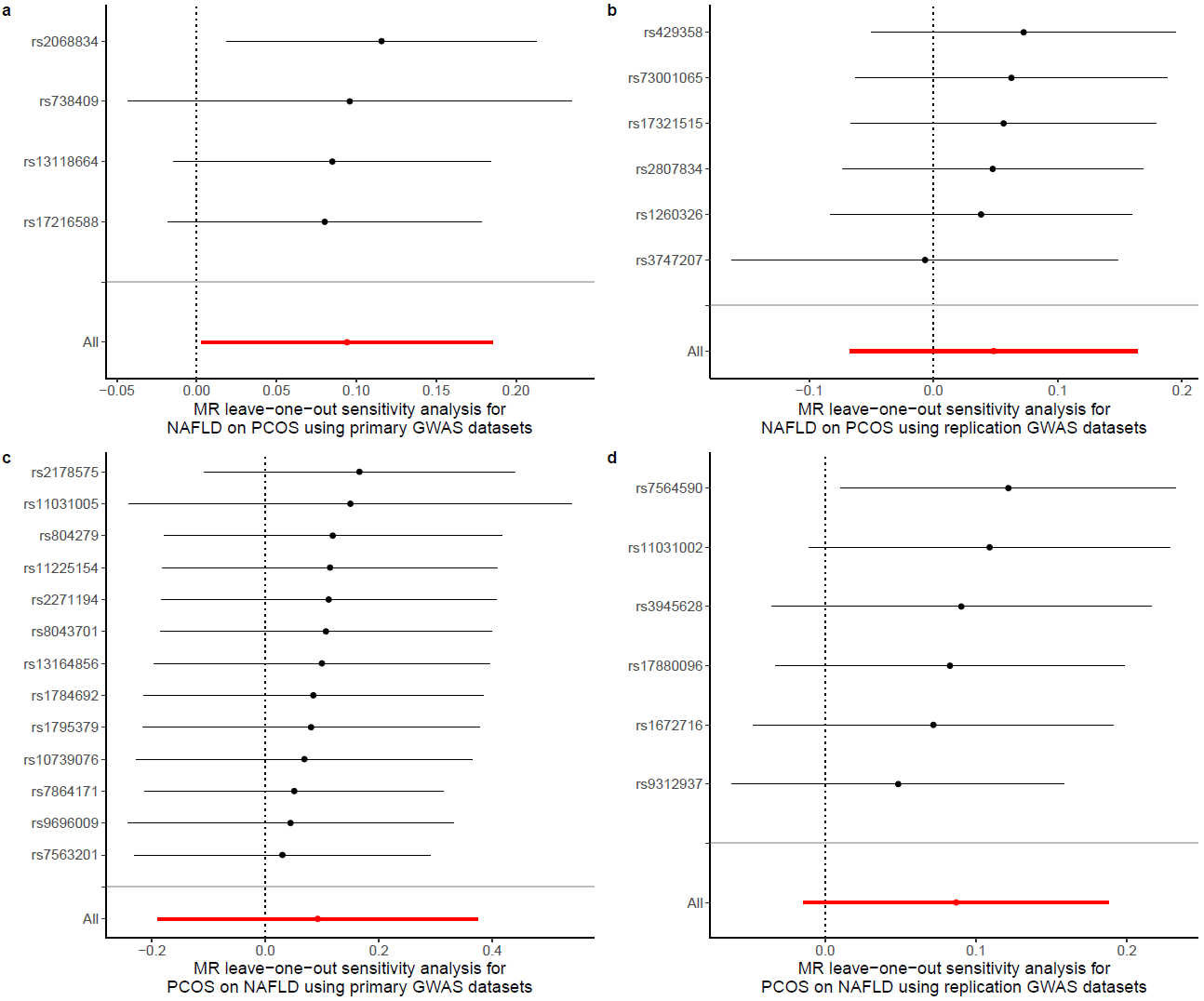


# **Fig S3. Leave-one-out analysis results of MR causal effects between NAFLD and PCOS.**

Panel a. Leave-one-out analysis for NAFLD on PCOS using primary GWAS datasets. Panel b. Leave-one-out analysis for NAFLD on PCOS using replication GWAS datasets. Panel c. Leave-one-out analysis for PCOS on NAFLD using primary GWAS datasets. Panel d. Leave-one-out analysis for PCOS on NAFLD using replication GWAS datasets.

Abbreviations: GWAS, genome-wide association study; MR, Mendelian randomization; NAFLD, non-alcoholic fatty liver disease; PCOS, polycystic ovary syndrome.


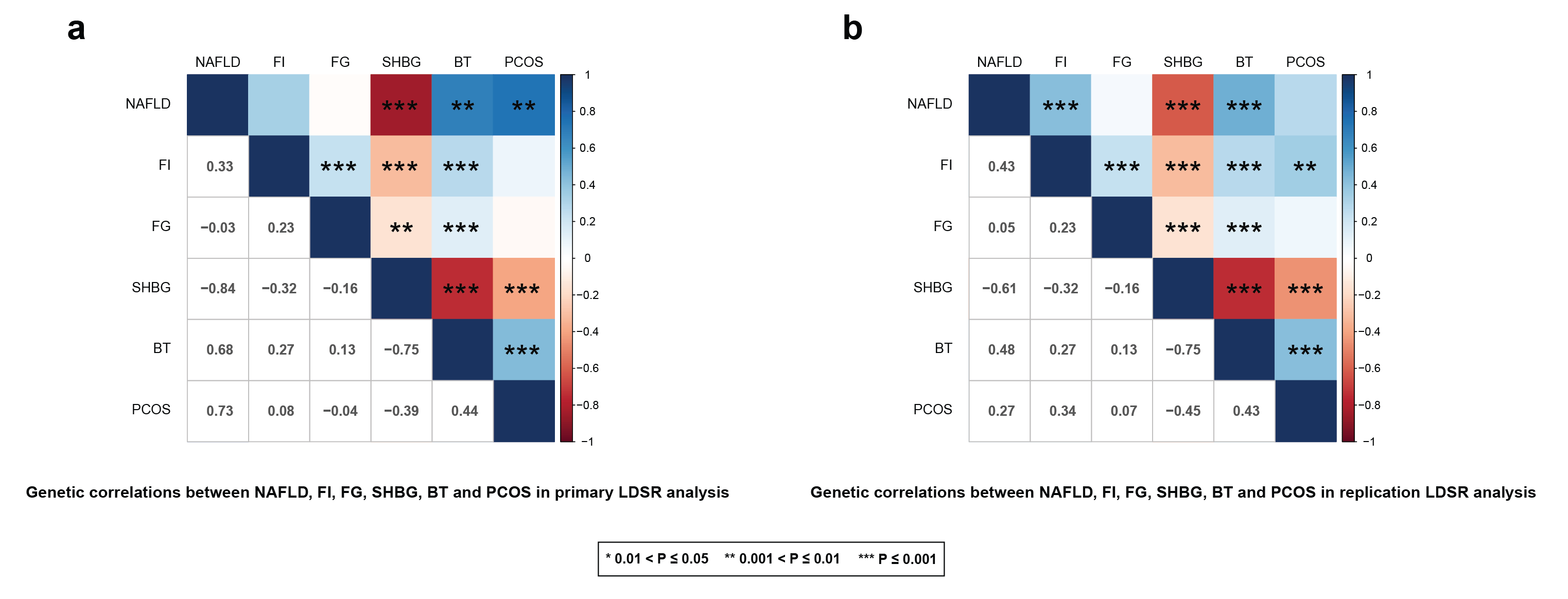


# **Fig S4. Genetic correlations between NAFLD, FI, FG, SHBG, BT, and PCOS in LDSR analysis.**

Panel a. Genetic correlations between NAFLD, FI, FG, SHBG, BT, and PCOS in primary LDSR analysis. Panel b. Genetic correlations between NAFLD, FI, FG, SHBG, BT, and PCOS in replication LDSR analysis.

The genetic correlations between each trait were presented in the lower left part of each panel.

Abbreviations: BT, bioavailable testosterone; FG, fasting glucose; FI, fasting insulin; LDSR, linkage disequilibrium score regression; NAFLD, non-alcoholic fatty liver disease; PCOS, polycystic ovary syndrome; SHBG, sex hormone-binding globulin.


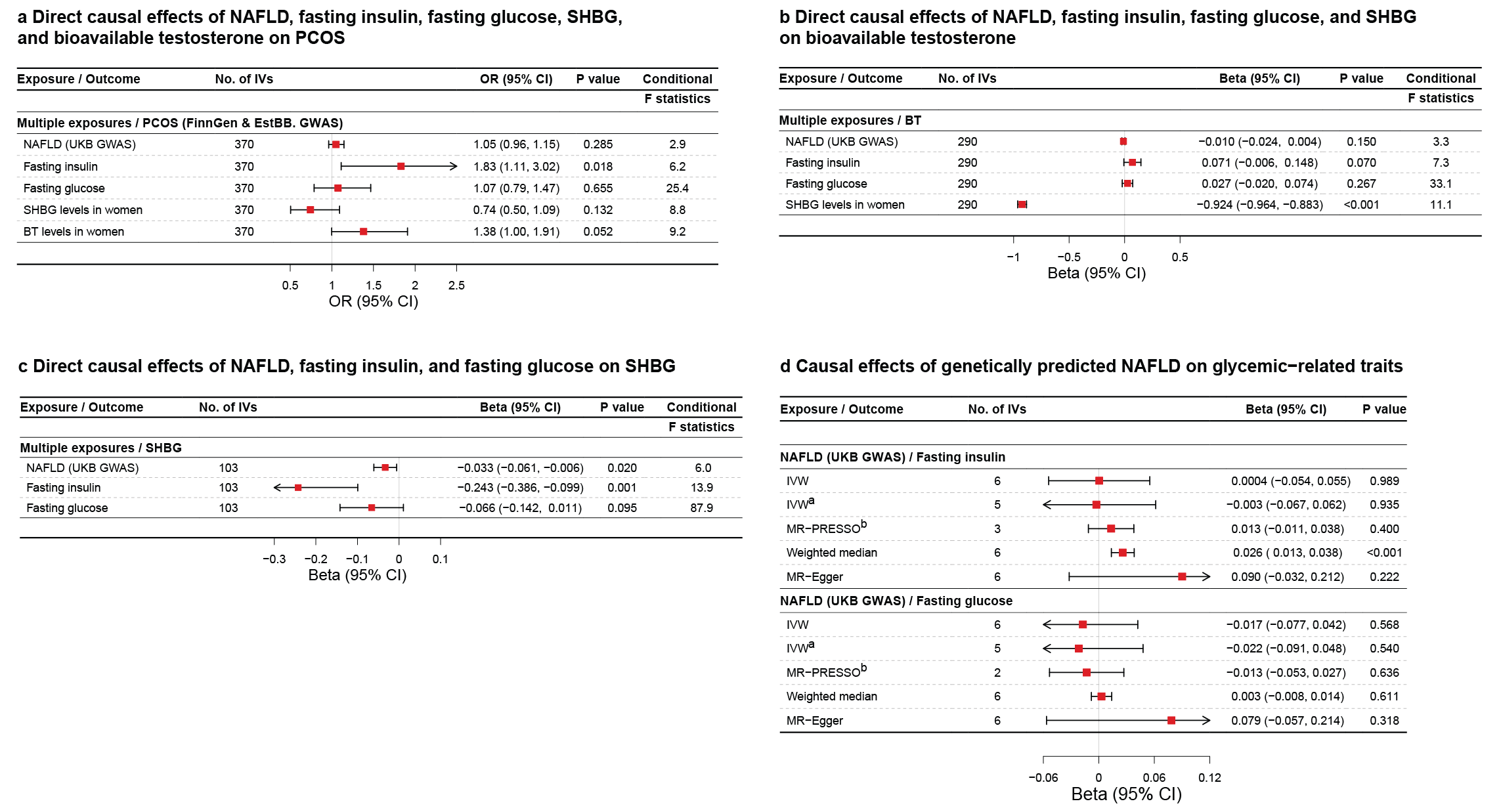


# **Fig S5.** **Results of step-wise MR mediation analysis for causal associations between NAFLD, glycemic-related traits, sex hormones, and PCOS using replication GWAS datasets.**

Panel a. Direct causal effects of NAFLD, glycemic-related traits, and sex hormones on PCOS. Panel b. Direct causal effects of NAFLD, glycemic-related traits, SHBG on BT. Panel c. Direct causal effects of NAFLD, glycemic-related traits on SHBG. Panel d. Causal effects of NAFLD on glycemic-related traits. MR-PRESSO analysis was not applicable to estimate the causal effect of NAFLD on fasting insulin and fasting glucose due to small number of genetic instruments used. MVMR analyses were conducted after excluding the obesity-related genetic variants.

a. A secondary IVW analysis was conducted after excluding rs429358 due to its genome-wide significant association with obesity.

b. Outlying genetic instruments were excluded in the corrected MR-PRESSO analysis.

Abbreviations: BT, bioavailable testosterone; IVs, instrumental variables; IVW, inverse-variance weighted; MVMR, multivariable Mendelian randomization; NAFLD, non-alcoholic fatty liver disease; PCOS, polycystic ovary syndrome; SHBG, sex hormone-binding globulin.


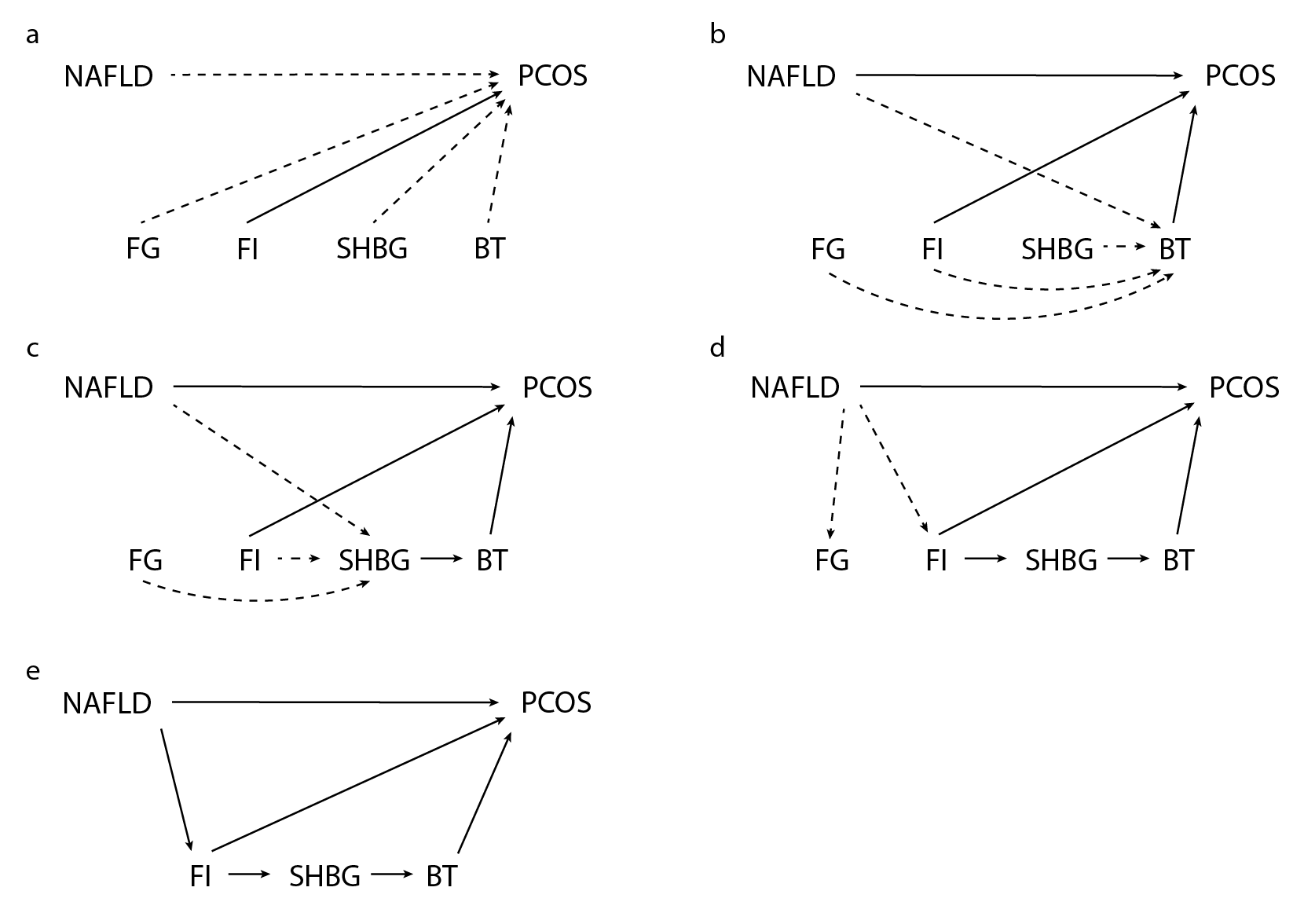


# **Fig S6.** **The overview of the step-wise MR mediation analysis between NAFLD and PCOS via glycemic-related traits and sex hormones.**

Panel a. MVMR analysis of NAFLD, glycemic-related traits, and sex hormones on PCOS. Panel b. MVMR analysis of NAFLD, glycemic-related traits, and serum SHBG levels on serum bioavailable testosterone levels. Panel c. MVMR analysis of NAFLD and glycemic-related traits on serum SHBG levels. Panel d. Two-sample MR analysis of NAFLD on glycemic-related traits. Panel e. The causal pathways tested in the present study between NAFLD and PCOS via glycemic-related traits and sex hormones. The dashed black lines with arrows represent the direct causal effects being tested for within each step using the MVMR analysis approach. The solid black lines with arrows represent the significant direct causal effects tested in the previous steps.

Abbreviations: BT, bioavailable testosterone; FG, fasting glucose; FI, fasting insulin; NAFLD, non-alcoholic fatty liver disease; PCOS, polycystic ovary syndrome; SHBG, sex hormone-binding globulin.


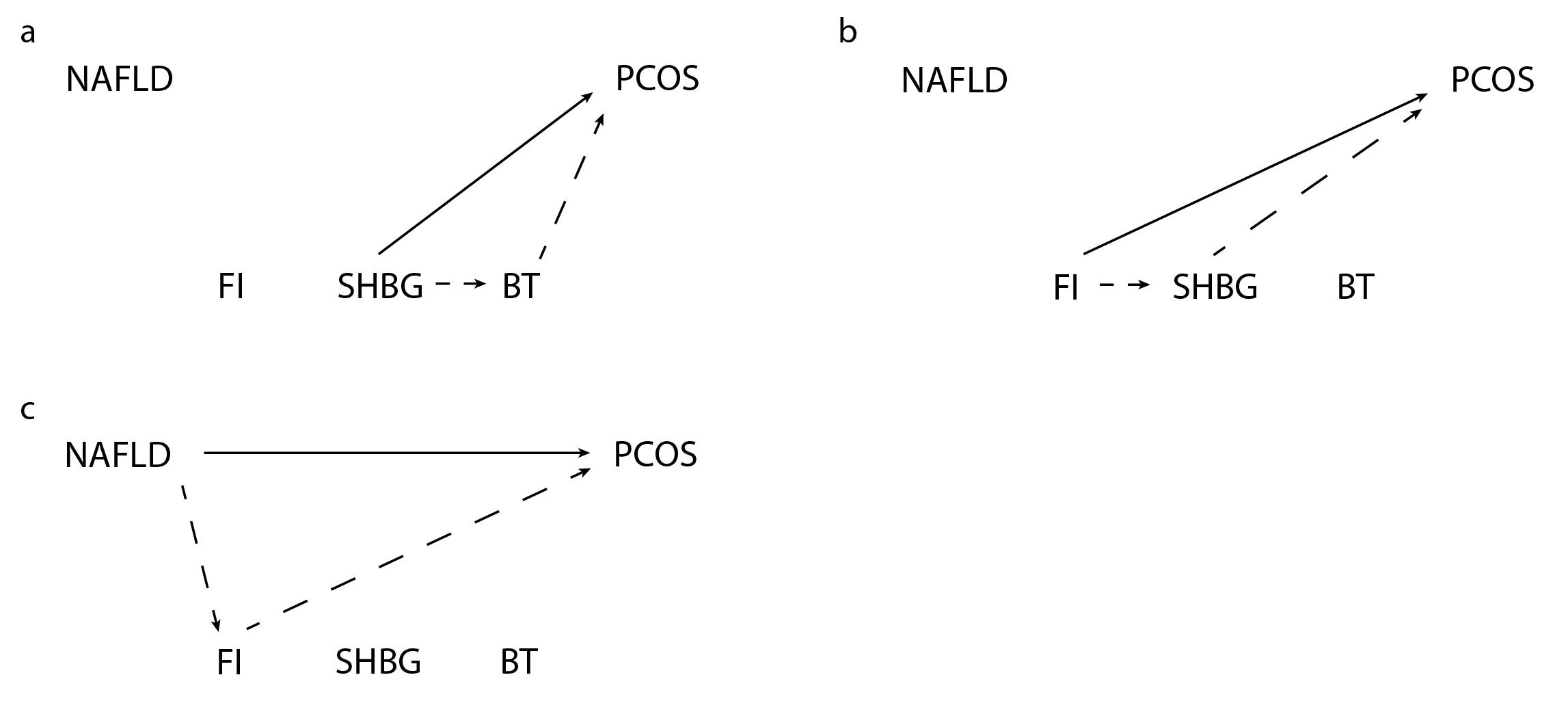


# **Fig S7. A schematic diagram of calculating the indirect causal effect of NAFLD on PCOS via fasting insulin and sex hormones.**

Panel a. The indirect causal effect of SHBG on PCOS was calculated based on the causal effects of SHBG on BT and BT on PCOS. Panel b. The indirect causal effect of FI on PCOS was calculated based on the causal effects of FI on SHBG and SHBG on PCOS. Panel c. The indirect causal effect of NAFLD on PCOS was calculated based on the causal effects of NAFLD on FI and FI on PCOS.

The dashed black lines with arrows represent the direct or indirect causal effects of the exposure on the mediator or the mediator on the outcome. The solid black lines with arrows represent the indirect causal effect between the exposure and the outcome via the mediator (dashed black lines) which were calculated using the two-step MR method.

Abbreviations: BT, bioavailable testosterone; FG, fasting glucose; FI, fasting insulin; NAFLD, non-alcoholic fatty liver disease; PCOS, polycystic ovary syndrome; SHBG, sex hormone-binding globulin.


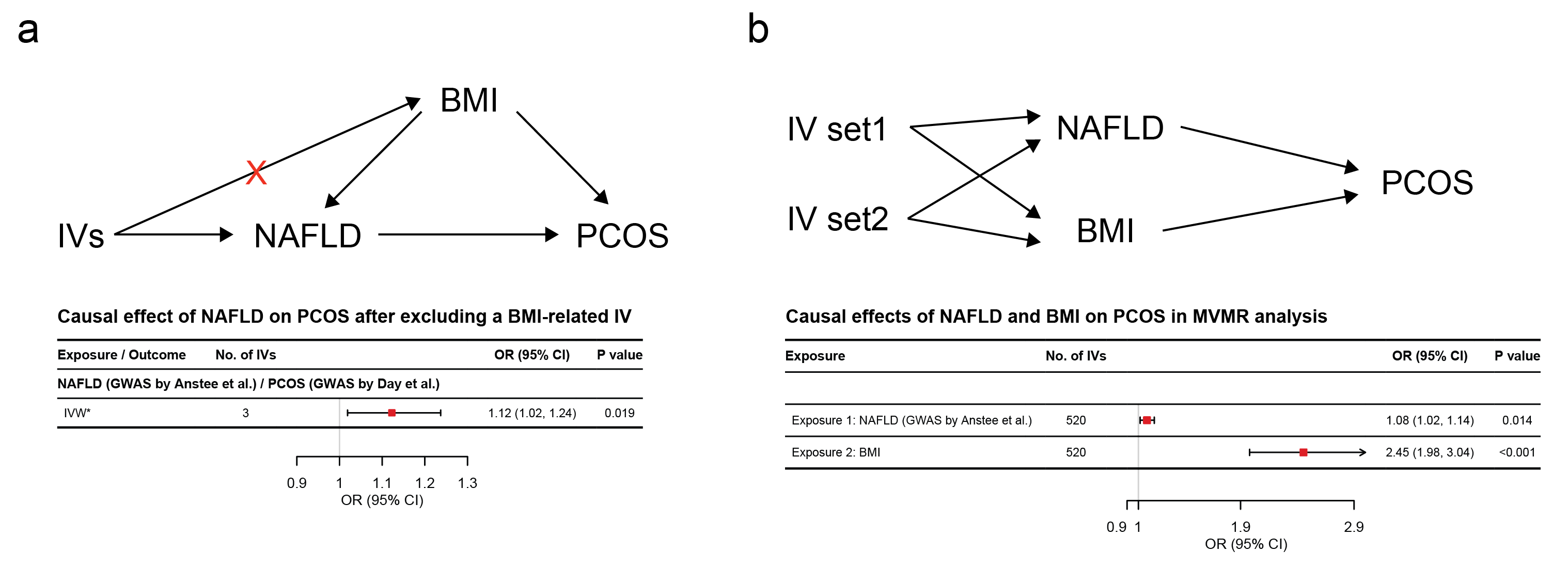


# **Fig S8. Causal effect of NAFLD on PCOS using two-sample MR after excluding BMI-related IV and using MVMR.**

Panel a. The causal effect of NAFLD on PCOS after excluding a BMI-related IV. Panel b. Causal effects of NAFLD and BMI on PCOS in MVMR analysis.

* rs2068834 was excluded from the sensitivity analysis due to its genome-wide significant association with obesity.

Abbreviations: BMI, body mass index; IVs, instrumental variables; IVW, inverse-variance weighted; MVMR, multivariable Mendelian randomization; NAFLD, non-alcoholic fatty liver disease; PCOS, polycystic ovary syndrome.
